# The Cerebellum Gets Social: Evidence from an Exploratory Study of Cerebellar, Neurodevelopmental, and Psychiatric Disorders

**DOI:** 10.3390/biomedicines11020309

**Published:** 2023-01-22

**Authors:** Giusy Olivito, Libera Siciliano, Silvia Clausi, Michela Lupo, Roberto Baiocco, Andrea Gragnani, Marco Saettoni, Roberto Delle Chiaie, Fiorenzo Laghi, Maria Leggio

**Affiliations:** 1 Department of Psychology, Sapienza University of Rome, 00185 Rome, Italy; 2 Ataxia Laboratory, Fondazione Santa Lucia IRCCS, 00179 Rome, Italy; 3 Klinikos Center for Psychodiagnostics and Psychotherapy, Viale delle Milizie 38, 00192 Roma, Italy; 4 Servizio di Tutela della Salute Mentale e Riabilitazione dell’Età Evolutiva ASL, Roma 2, 00145 Rome, Italy; 5 Department of Developmental and Social Psychology, Sapienza University of Rome, 00185 Roma, Italy; 6 Scuola di Psicoterapia Cognitiva SPC, 58100 Grosseto, Italy; 7 Associazione Psicologia Cognitiva (APC)/Scuola di Psicoterapia Cognitiva (SPC), 00185 Rome, Italy; 8 Unità Funzionale Salute Mentale Adulti ASL Toscana Nord-Ovest Valle del Serchio, 56121 Pisa, Italy; 9 Department of Neuroscience and Mental Health–Policlinico Umberto I Hospital, Sapienza University of Rome, 00161 Rome, Italy

**Keywords:** prediction, cerebro-cerebellar circuits, cerebellar atrophy, social cognition, cerebellar pathologies, autism, bipolar disorders, voxel-based morphometry

## Abstract

Social prediction is a key feature of social cognition (SC), a function in which the modulating role of the cerebellum is recognized. Accordingly, cerebellar alterations are reported in cerebellar pathologies, neurodevelopmental disorders, and psychiatric conditions that show SC deficits. Nevertheless, to date, no study has directly compared populations representative of these three conditions with respect to SC and cerebellar alterations. Therefore, the present exploratory study aimed to compare the SC profiles of individuals with cerebellar neurodegenerative disorders (CB), autism (ASD), bipolar disorder type 2 (BD2), or healthy subjects (HS) using a battery of social tests requiring different degrees of prediction processing. The patterns of cerebellar gray matter (GM) alterations were compared among the groups using voxel-based morphometry. Compared to HS, the clinical groups showed common SC deficits in tasks involving a moderate to high level of prediction. The behavioral results of the clinical groups are consistent with the presence of overlapping GM reduction in cerebellar right Crus II, an area notably involved in complex social processing and prediction. Although exploratory and preliminary, these results deepen the cerebellar role in social prediction and highlight the transdiagnostic value of the cerebellum in social functioning and prediction in pathologies of different aetiologies, forecasting novel possibilities for shared interventions.

## 1. Introduction

The human brain is predisposed to build contingent associations between signals and outcomes of social understanding and interaction [1]. The computation of probabilities based on prior experiences allows humans to make inferences and predictions about the most likely social outcome [1]. Considering social prediction as involving the processing of a multitude of socially relevant stimuli (e.g., social perception, social cognition, empathy, and social decision-making), significant overlaps between the “social” brain and the “predictive” brain have been reported for many areas, including the dorsal and medial prefrontal cortex (PFC), the temporoparietal junction, the precuneus, the amygdala, the ventral striatum, and the premotor and somatosensory cortices [2]. 

Although underestimated until a few years ago, the cerebellum is now acknowledged to contribute to affective and high-order functions and to form part of composite neural networks that subtend social prediction [3,4,5,6,7,8,9,10,11,12,13]. Such recognition is rooted in the well-established role of the cerebellum as a key node in the neural system engaged for creating and adapting internal models for sensorimotor control [14,15,16]. In this system, the cerebellum operates by creating internal models of anticipated sensory outcomes of the motor commands that it receives and combines with exteroceptive and proprioceptive sensory inputs [17]. In this way, the cerebellum generates outcome prediction based on forward models, detects deviations from these outcomes, and ultimately emits corrective signals that are used to adjust upcoming predictions regarding future movements, guaranteeing skillful sensorimotor control [14,15]. 

The demonstration that cerebellar circuitry is uniform throughout the entire cerebellar cortex and that the cerebellum has numerous connections with many areas of the cerebral cortex encouraged the concept that the cerebellum might operate in the optimization of various functions in much the same way in which it acts in motor control [18]. This proposed role of cerebellar functioning has also been extended to social cognition (SC), since the recognition of prediction errors during social interaction is useful in adjusting socially predictable sequences and assuring adaptive social behaviors in the future [10,19]. Certain areas of the posterior cerebellum are mostly engaged in processing social sequences and signals [13,20], in handling situations that require detection of prediction errors regarding aversive events [21], in detecting violations from social norms [22] or in predicting events that may occur in the future [15,23]. The involvement of the posterior cerebellum in social and predictive processing is further supported by the massive amount of evidence regarding the structural and functional connections between areas in this portion of the cerebellum and the areas reported above as part of both the “social brain” and the “predictive brain” [19,24,25,26,27,28,29]. It is commonly assumed that functional modulation by the cerebellum on the projection areas in the cerebral cortex might be crucial for optimizing and revising high-order functions and that this mainly contributes to the generation or fine-tuning of new experience-based internal models [15]. The existence of a domain-general supramodal operational mode of the cerebellum explains the occurrence of a wide variety of cognitive, affective, and social symptoms in patients with cerebellar disease [19,30,31,32,33,34,35]. 

Research on patients with cerebellar diseases or abnormalities has reported deficits in multifaceted aspects of social cognitive domains such as emotion recognition, social judgements, intention prediction, and identifying others’ beliefs [11,19,20,31,32,35,36]. Inadequate behavioral outcomes in these domains are underpinned by structural impairment in Crus I-II, the cerebellar lobules that are activated during SC tasks, and altered functional exchange of information between these cerebellar areas and areas of the social brain such as the PFC, the TPJ, and the precuneus [19,26,30,37]. It has been suggested that when cerebellar damage occurs, the proper exchange of information in cerebellar-cerebral loops and, thus, the constant comparisons between external stimuli and internal models are affected, leading patients with cerebellar damage that fails to recognize social prediction errors [19,30]. 

Impairments in the above-mentioned regions of the cerebellum have also been associated with the onset of emotional and social cognitive deficits in a variety of neurodevelopmental and psychiatric disorders such as autism spectrum disorder, schizophrenia, depression, and bipolar disorders [30,38,39,40,41,42,43,44]. Some studies refer to autism as a disorder of prediction in the sense that compromised prediction impedes social and communication skills, causing individuals with autism to inhabit overwhelming and confusing social environments in which events mainly occur in an unpredictable way [45,46,47,48,49]. The neural underpinning of affected predictive abilities and social skills in autism are ascribed by some authors to impaired modulation by the posterior cerebellum on cerebral projection areas involved in social functions [30,50].Consequently, altered functional connectivity between Crus II and areas of the brain that mediate social behavior has been described in individuals with autism [50,51]. Interestingly, similarities in theory of mind profiles have been found when the SC performances of patients with cerebellar degenerative disease are compared with those of individuals with autism spectrum disorder with no language or intellectual impairment, and these similarities are likely caused by shared gray matter (GM) reduction in Crus II of the cerebellum [30]. The prediction-based theories about social and psychotic symptoms are consistent with the “dysmetria of thought” hypothesis described by Schmahmann and colleagues [52] According to this hypothesis, cerebellum-related cognitive and affective deficits mirror diminished (hypometric) or exaggerated (hypermetric) responses to the internal and/or external environment, causing aberrant prediction-based “mindworld synchronization” [15,52,53,54].

In this sense, cerebellar alterations might also explain increased or decreased responses to the external or internal environment and, hence, the poor mood homeostasis with alternation of depressive and manic phases that is typical of bipolar disorders (BDs). BDs are characterized by episodes of depression and episodes of mania (BD type 1-BD1) or hypomania (BD type 2-BD2) with interepisodic euthymic/remission phases [55,56]. While predictive processing has been related to cerebellar functioning in autism and schizophrenia, these issues have not yet been investigated in BD. However, the occurrence of SC deficits in individuals with BD has been reported [57,58,59,60], and the hypothesis that the cerebellum might play a role in the neuropathophysiological mechanisms of BDs has been explored [43,44,61,62]. Neuroimaging studies conducted in the last twenty years have revealed the presence of cerebellar alterations in patients with BDs [43,44,62,63,64,65,66]. Interestingly, in a study that compared cerebellar atrophy patterns in patients with cerebellar neurodegenerative disease with those in patients in the euthymic phase of BD, areas of overlapping GM reduction were found in lobule V, right Crus I and bilateral crus II [62]. In addition, in a recent study, a significant pattern of cerebellar atrophy involving the cerebellum was found in euthymic BD1 and BD2 patients compared to healthy controls, with more diffuse GM reduction in BD2 patients as well as more widespread ToM deficiencies, suggesting that BD2 patients are more impaired than BD1 patients in their mentalizing abilities [44]. 

It is worth noting that although these clinical conditions are conceptualized as distinct diagnostic entities based on their peculiar clinical status, the actual literature on the psychopathological manifestations of ASD and BD have proven the existence of shared clinical features [67], neural networks [68], genetic architecture [69,70], and aetiological risk factors [71,72]. Accordingly, a large body of studies has reported high rates of comorbidity between ASD and BD [73,74,75,76]. In BD, the presence of autistic traits has been frequently reported [75,77] impacting on functional impairment, rates of suicidality and severity of depressive symptoms [75]. In ASD, suicidal tendencies are commonly documented, specifically in high-functioning ASD individuals [78,79]. Interestingly, both affective and social impairments have been reported in patients with cerebellar diseases, many of which overlap with symptoms typical of autism or mood disorders [30,52,80,81]. Consequently, recent research studies have highlighted the importance of focusing on transdiagnostic dimensional features shared between categorical disorders [68,82,83]. In this view, the cerebellum might assume a central role since cerebellar structural alterations have been stated to represent a shared risk factor for diverse psychopathologies, due to its core role to modulate cerebral activity and to optimize and to make prediction about information in various domains [82,83]. 

All in all, considering the above-mentioned state-of-the-art research on the role of the cerebellum in SC deficits that occur in different pathological conditions such as cerebellar disease, ASD, and BDs, we speculated that these clinical populations share cerebellar alterations as well as social cognitive deficits that might be explained in terms of prediction failure, and in turn likely caused by an altered cerebro-cerebellar internal model system. To propose and explore these hypotheses, in the present exploratory study, the SC abilities of individuals affected by degenerative cerebellar atrophy, those of individuals with ASD who exhibit no language or intellectual impairment, and those of individuals with BD2 in the euthymic phase were compared. An ad hoc SC battery employing tasks that require different levels of prediction was used to examine automatic processes and more complex aspects of social cognition. A morpho-volumetric analysis was also performed by using a voxel-based morphometry (VBM) to characterize the shared and distinctive cerebellar structural changes in these individuals.

All the participants were previously engaged for other studies conducted by our research group in which we aimed at comparing CB and ASD ToM profile, and CB, BD1, and BD2 ToM profile, respectively [30,44]. Nevertheless, this is the first study aimed at comparing the SC profile also between ASD and BD2, making use of a composite battery of social cognition tests that allows us to examine the ability to attribute emotions to others other than ToM.

## 2. Materials and Methods

### 2.1. Participants

The present study included 32 patients with cerebellar neurodegenerative disorders (CB) [(F/M: 17/15; age (mean/SD): 46.71/11.03; education (mean/SD): 13.4/3.45)], 21 adults with autism spectrum disorder (ASD) [(F/M: 10/11; age (mean/SD): 26.85/8.66; education (mean/SD): 13.76/1.72)], and 13 patients affected by bipolar disorder type 2 (BD-2) [(F/M: 6/7; age (mean/SD): 45.07/11.22; education (mean/SD): 14.53/3.79)]. All of these individuals were previously enrolled in other studies conducted by our group [30,44].

The CB patients suffered from neurodegenerative diseases of different aetiologies. CB patients were enrolled from patients admitted to the IRCCS Santa Lucia Foundation rehabilitation hospital. When recruited, all CB patients had been diagnosed for more than 6 months. T2-weighted MRI scans were visually inspected to exclude the presence of extracerebellar lesions. The inclusion of patients was ensued when no history of extracerebellar neurologic pathology or psychiatric disorders was reported and when patients had average intellectual levels.

The ASD subjects had no language impairment or intellectual impairment based on the Diagnostic and Statistical Manual of Mental Disorders, Fifth Edition (DSM-5) criteria [84] and were enrolled through the assistance of Gruppo Asperger Onlus, Spazio Asperger Onlus, CulturAutismo Onlus, Cooperativa Giuseppe Garibaldi, and ANGSA (Associazione Nazionale Genitori Soggetti Autistici).

The BD2 patients were enrolled from the bipolar disorder outpatient ward of the Department of Psychiatry, Policlinico Umberto I Hospital. All of them met the criteria set forth in the DSM-5 for BD, according to a diagnostic assessment performed using the Italian version of the Structured Clinical Interview for DSM-5—Clinician Version (SCID-5-CV) [85]. All patients had been euthymic for at least three months. The euthymic phase was confirmed by administration of the Hamilton Depression Rating Scale (HDRS score < 10) [86] and the Young Mania Rating Scale (YMRS score < 13) [87]. The pharmacotherapy of BD2 is reported in Table S1.

Specific control groups were enrolled for the SC and MRI examinations.

Fifty-seven healthy subjects (HS-SC) [(F/M: 33/24; age (mean/SD): 36.49/13.15; education (mean/SD): 13.78/2.73)] of normal intellectual level (>18.96) [88] and no history of neurological disorders or psychiatric illness served as the control group for the SC comparison. One-way ANOVA was conducted to compare the experimental groups enrolled for SC testing with respect to sex, age, and education. No significant difference was detected in sex distribution (F = 0.331, *p* = 0.803) or in education (F = 0.227, *p* = 0.797), whereas a significant difference was found in age (F = 13.602, *p* = 0.000).

Two control groups of healthy subjects (HS-MRI-1 and HS-MRI-2) were used in the MRI analysis. Retrospective MRI data were collected from these participants between 2014 and 2019 at the Neuroimaging Laboratory of Santa Lucia Foundation. The HS-MRI-1 group, which was used as a control for the CB and BD2 patients, included 39 healthy subjects [(M/F = 175/22; age (mean/SD) = 44.9/14.5)]. Statistical analysis revealed no significant differences between the CB and HS-MRI-1 groups in age (*t* test: *t* = −0.60, *p* = 0.54) or sex distribution (chi-square: χ^2^ = 0.00, *p* = 0.98) or between BD2 and HS-MRI-1 (*t* test for age: t = 0.19, *p* = 0.84; chi-square for sex: χ^2^ = 0.41, *p* = 0.52). The HS-MRI-2 group, which was used as a control for the ASD individuals, consisted of younger healthy subjects [(M/F = 21/10; age (mean/SD) = 26.1/5.23)]. Again, statistical analysis revealed no significant differences between the ASD and HS-MRI-2 groups in age (*t* test: t = −0.14, *p* = 0.88) or sex distribution (chi-square: χ^2^ = 3.17, *p* = 0.07).

The individuals in the CB, ASD and BD2 groups underwent neurological assessment and evaluation of cerebellar motor deficits by means of the International Cooperative Ataxia Rating Scale (ICARS) [89]. The total score on this scale varies from 0 (indicative of absence of motor deficits) to 100 (greatest extent of motor deficits). HS-SC, CB, ASD, and BD-II participants also underwent the Wechsler Adult Intelligence Scale-Revised (WAIS-R) [90,91] or the Raven progressive matrices test [88] to prove the presence of a normal intellectual level. The Autism Spectrum Quotient was used to verify the presence of autism traits in the ASD and HS-SC groups [92]. All CB, ASD, and BD2 individuals underwent the ToM assessment and MRI protocols. Table 1 reports participants’ demographic characteristics and the results achieved in the screening phase.

This research study was approved by the Ethics Committee of Santa Lucia Foundation, according to the principles expressed in the Declaration of Helsinki. Written informed consent was obtained from each subject.

### 2.2. Social Cognition Assessment

To examine social cognition ability, we employed an ad hoc battery of tests that evaluated different degrees of prediction processing.

The Emotion Attribution test (EA) [93,94] was used to investigate the capacity to infer others’ emotions in a social context. The test comprised fifty-eight short stories representing emotional circumstances that would be expected to elicit happiness, sadness, fear, anger, disgust, embarrassment, or envy. The participants were asked to describe the main character’s feelings in that situation using a one-word description. This test requires a low level of social prediction since it implies univocal sequences of routine emotional events and includes explicit essential contextual information. The proper processing of the emotional impact is based on having a coherent expectation about the social interaction. Responses were scored 1 if accurate and 0 if wrong.

The Reading the Mind in the Eyes Test (RMET) [95,96], one of the tests that is most used to assess the automatic lower-level of mentalizing processes, requires individuals to attribute a person’s mental state by observing that individual’s gaze irrespective of the context. The test evaluates the ability to decode another’s state of mind based on observable information that is instantaneously accessible. The participant is required to match the semantic definition of a complex mental state (e.g., cautious, sarcastic, bewildered) with a picture of the eye region of an individual depicted in black-and-white photographs. This test requires a moderate level of social prediction since it requires the ability to identify the underlying intention and complex mental state from eye expression alone, with no other cues available. The test consists of 36 photographs of actors’ eyes; for each photograph, the participant must choose which of four words best describes the mental state conveyed by the male or female actor’s eyes. Responses were scored 1 if accurate and 0 if wrong.

The Faux Pas test (FP) [97,98] was used to assess an advanced component of ToM, namely, the ability to make complex assumptions about another person’s mental states. The FP test included 10 stories (“faux pas” stories) in which a social “faux pas” occurred and 10 control stories in which no social faux pas occurred (“no-faux pas” stories). A social faux pas occurs when a person says something inappropriate without intention of hurting others but without considering that the listener might be upset by what has been said. Thus, recognizing a social faux pas requires understanding of both the mistaken or false belief and the emotional effect on the listener. Accordingly, the FP test allows us to disentangle the cognitive component (questions 1–5) from the affective component (question 6) of ToM. When the faux pas is identified, questions 2–6 are asked to deepen the participant’s understanding of the mental and emotional states of the protagonists. When a social situation occurs, agents must compare others’ ongoing behaviors with models of actions they have internalized in the past, allowing them to make predictions based on learned social standards. In this way, the FP test allows us to analyze situations in which social conditions require a different degree of predictive capacity. Indeed, when a faux pas occurs, the social events are ambiguous and unexpected and demand a high level of prediction to be properly understood since a consistent comparison between social anticipation and live events, as well as adjustments for errors, are required. In contrast, the “no-faux pas” stories depict univocal and expected social events that require a reduced predictive capacity to be adequately understood since they mirror situations that correspond to previously learned internal models of appropriate social behaviors. Each correct answer to the questions about the faux pas stories was assigned a score of 1, and each incorrect answer was assigned a score of 0; the maximum score was 6. When the participant accurately identified the absence of a faux pas in one of the “no-faux pas” stories, he or she received a score of 2. For each story, two control questions were asked to determine whether the participant understood the story. Each answer was scored 1 when correct and 0 when wrong.

### 2.3. Data Analyses

One-way multivariate analysis of covariance (MANCOVA) with age as a covariate was conducted to compare each variable in a between-groups design. Pairwise comparisons with Bonferroni adjustment were performed when significant differences were detected (*p* < 0.05). Partial eta-squared values were considered for measuring effect size. Cohen’s guidelines [99] were used to interpret the results in terms of small (0.01), medium (0.06), and large (0.14) effect sizes.

### 2.4. Voxel-Based Morphometry

Voxel-based morphometry (VBM) was used to identify differences in regional cerebellar volume in the CB, ASD and BD2 groups compared to their respective control groups. The cerebellum was preprocessed individually using the Spatially Unbiased Infratentorial Template (SUIT) toolbox [100] implemented in statistical parametric mapping [Wellcome Department of Imaging Neuroscience; SPM-8 (http://www.fil.ion.ucl.ac.uk/spm/) accessed on 12 December 2022]. The detailed preprocessing steps are the same as those described in our previous papers [30,62]. A voxelwise two-sample T test was performed to assess differences in regional GM cerebellar volumes between each group (CB, ASD, BD2) and its respective control group. No nuisance variables were entered. Additionally, one-way ANOVA was performed to compare patterns of reduction in cerebellar GM among the CB, ASD and BD2 groups with age entered as a variable of no interest. For both analyses, the cerebellum was entered as an explicit inclusion mask. The results were considered significant at *p* values < 0.05 after FWE cluster-level correction.

## 3. Results

### 3.1. Social Cognition Profile

MANCOVA revealed a statistically significant difference among the four groups with respect to the combined dependent variables after controlling for age [F (15, 301) = 5.147, *p* < 0.000, Wilks’ Λ = 0.533, partial η^2^ = 0.189].

**Figure 1:**
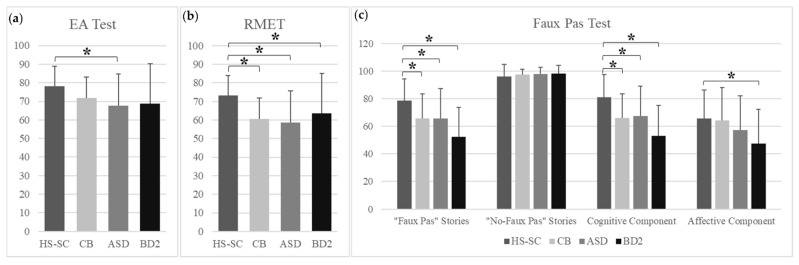
Results of the Social Cognition battery. (**a**) EA Test results. Scores are reported as the percentage of the overall number of accurate answers (max = 58). (**b**) RMET results. Scores are reported as the percentage of the overall number of accurate answers (max = 36). (**c**) Faux Pas Test results. Scores are reported as the percentage of the overall number of accurate answers given to all the questions of the faux pas stories (max = 60) and no-faux pas stories (max = 20), to the questions evaluating the Cognitive Component (max = 50) and to those evaluating the Affective component (max = 10). For each group, mean and standard deviation of the percentage of accuracy are reported, with 0% representing completely incorrect responses and 100% representing completely accurate responses; * *p* < 0.05. Abbreviations: HS-SC, healthy subjects; CB, degenerative cerebellar damage; ASD, autism spectrum disorders; BD2, bipolar disorders type 2.

A significant group effect was found in the EA test [F (3, 113) = 5.794; *p* = 0.001, ηp2 = 0.13]. The pairwise comparisons with a Bonferroni adjustment revealed that the ASD group had significantly lower scores than the control group on this test (ASD vs. HS-SC: *p* = 0.002), while no significant differences were found in the other pairwise comparisons between the groups (Figure 1a).

A significant group effect was found in the RMET [F (3, 113) = 10.788; *p* = 0.000, ηp^2^ = 0.22]. Pairwise comparisons with Bonferroni adjustment revealed that all three clinical groups had significantly lower scores than the control group (CB vs. HS-SC: *p* = 0.001; ASD vs. HS-SC: *p* = 0.000; BD vs. HS-SC = *p* = 0.054), while no significant differences were found among the clinical groups (Figure 1b).

Regarding the FP test, a significant group effect was found in the faux pas total [F (3, 113) = 8.831; *p* = 0.000, ηp^2^ = 0.19], cognitive [F (3, 113) = 10.058; *p* = 0.000, ηp^2^ = 0.21] and affective [F (3, 113) = 2.631; *p* = 0.054, ηp^2^ = 0.06] components of the faux pas test. No group effect was found in the no-faux pas total [F (3, 113) = 0.656; *p* = 0.581, ηp^2^ = 0.01]. Pairwise comparisons with Bonferroni adjustment revealed that all three clinical groups had significantly lower scores than the control group in the faux pas total (CB vs. HS-SC: *p* = 0.012; ASD vs. HS-SC: *p* = 0.000; BD2 vs. HS-SC: *p* = 0.054). All three clinical groups had significantly lower scores than the control group in the cognitive component of the faux pas test (CB vs. HS-SC: *p* = 0.003; ASD vs. HS-SC: *p* = 0.031; BD2 vs. HS-SC = *p* = 0.002). Regarding the affective component, the BD2 group had significantly lower scores than the control group (BD2 vs. HS-SC: *p* = 0.051). No significant differences were found for the other pairwise comparisons between the groups (Figure 1c).

The means and SDs of the scores obtained by each group in the tests are reported in Table 2. For detailed statistics, see Table S2 in the Supplementary Materials.

### 3.2. Voxel-Based Morphometry

**Figure 2:**
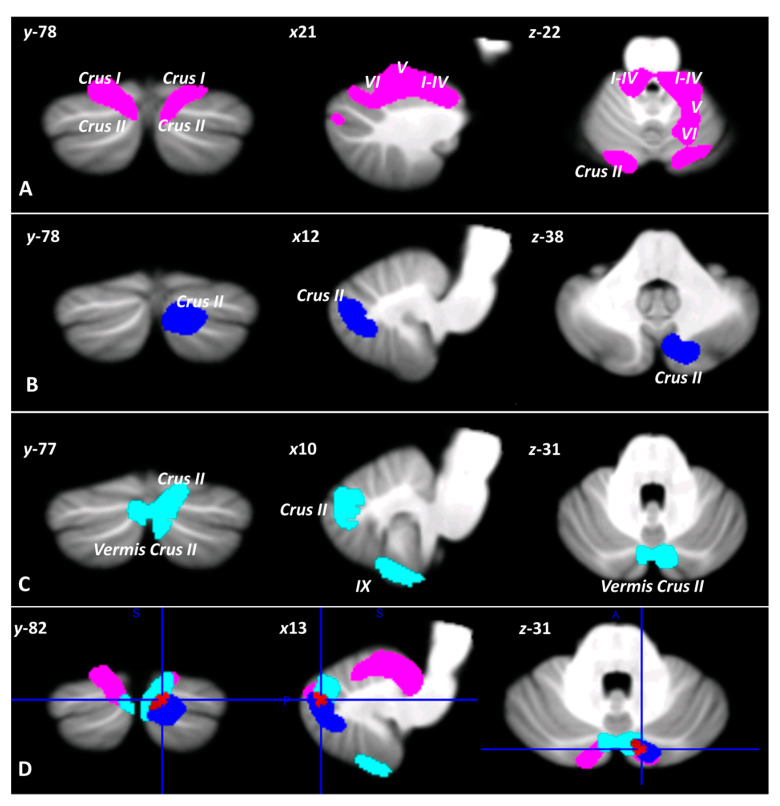
Voxel-wise two sample-*t* tests on cerebellar GM density. Cerebellar regions showing patterns of significantly reduced GM in CB (**A**), ASD (**B**), and BD2 (**C**) compared to respective control groups are reported and superimposed on the Spatially Unbiased Infratentorial Template (SUIT) [100] in coronal (y), axial (z) and sagittal (x) sections. The results were significant at *p*-values < 0.05 after familywise error (FWE) cluster-level correction. The region of overlapping cerebellar GM reduction in CB, ASD and BD2 is shown in red (**D**) superimposed on the SUIT. The images are presented according to neurological convention.

As shown by two-sample T tests, different patterns of regional decrease in cerebellar GM were found in the CB, ASD, and BD2 groups compared to the controls. Specifically, two large clusters of cerebellar GM reduction were found in CB patients; these clusters showed peak voxels in left lobules I–IV and left Crus II, extending to right lobules I–IV, bilateral lobule V, bilateral Crus I, and right Crus II (Figure 2A). In ASD individuals, a single cluster of reduced cerebellar GM was found with peak voxels in right Crus II, partially extending to right Crus I (Figure 2B). In BD2 patients, two clusters with reduced cerebellar GM were found; these clusters had peak voxels in right lobule IX and Crus II, extending to bilateral Crus I, left lobule IX and vermis Crus II (Figure 2C). Interestingly, a common pattern of cerebellar GM atrophy was evident in the right Crus II (Figure 2D). Detailed statistics for the two-sample T tests are reported in Table 3.

**Figure 3:**
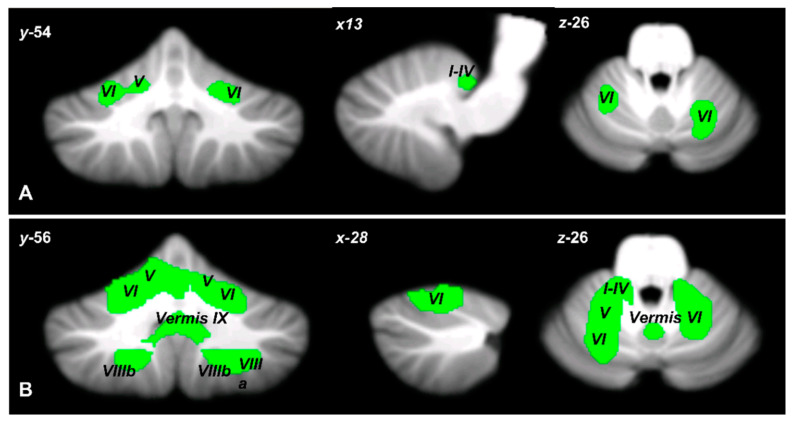
Voxel-wise ANOVA on cerebellar GM density. Cerebellar regions showing patterns of significantly reduced GM in CB compared to ASD (**A**) and BD2 (**B**) are reported and superimposed on the Spatially Unbiased Infratentorial Template (SUIT) [100] in coronal (y), axial (z), and sagittal (x) sections. The results were significant at *p*-values < 0.05 after familywise error (FWE) cluster-level correction. The images are presented according to neurological convention.

Finally, direct comparison of the CB, ASD, and BD2 populations revealed that CB patients differed significantly from ASD and BD2 patients in cerebellar GM atrophy. In particular, two large clusters of significant decreases in GM were found in CB patients compared to ASD individuals; these clusters had peak voxels in mostly motor cerebellar regions such as right lobules I–IV, V, VI, and left lobules V and VI (Figure 3A). Similarly, compared to BD2 patients, CB patients had one large cluster of significant GM decrease with peak voxels in mostly motor cerebellar regions such as right lobules V and VIIIb and left lobule VI, extending to left lobules I–IV, V, VIIIa and VIIIb, right lobules I–IV, VI, VIIIa, and vermis VI, VIIIa, VIIIb, and IX (Figure 3B). Detailed statistics of the between-group ANOVA are reported in Table 4.

## 4. Discussion

In recent years, the role of the cerebellum in the context of sensorimotor and cognitive predictive coding [14,15,101] has also been extended to the SC domain [10,19]. Indeed, predicting errors during social interactions allows us to anticipate predictable social events and consequently to modify behavior when these predictions are violated.

Social cognition deficits have been widely described in patients with cerebellar disorders [19,37] as well as in individuals with neurodevelopmental (i.e., ASD) [102,103,104] and psychiatric conditions (i.e., BD) [39,57], in which cerebellar alterations are also reported [30,43,44,50,62].

The mechanisms by which the cerebellum supports social functioning can be described in terms of the bidirectional anatomical connections between the cerebellum and areas of the limbic system and of the frontal and temporoparietal lobes known to be engaged for emotional control and for the processing of socially salient clues [27,33]. Specifically, the constant exchange of information between cerebellar modules and cortical projection regions provided by cerebellar-cerebral circuits might contribute to refine the predictive features of social behavior [15,19].

To further clarify this issue, in the present exploratory study we examined and compared SC ability in CB, ASD and euthymic BD2 individuals using well-known social cognitive tasks that require different level of anticipation and prediction. Furthermore, to identify common and distinctive patterns of cerebellar alterations that, in accordance with previous literature, might be related to impairment of social prediction ability, structural changes in the cerebellum were characterized in the three groups.

Compared to controls, CB, ASD, and BD2 patients showed common impairments in the automatic and more complex SC abilities that require a moderate to high level of prediction, as shown by significantly different RMET total scores and total and cognitive Faux Pas scores.

Overall, the present results show that impaired SC performance is observed in the three examined populations when sequential events occurred unexpectedly and ambiguously, thus demanding a continuous check between the actual event and the social anticipations, together with a significant degree of prediction [19]. Accordingly, to detect a “faux pas” (i.e., stories in which a protagonist says something inconvenient without realizing it) one is required to cognitively understand that a person has said something awkward against the conventional and predicted behavioral patterns, but also to predict the effect that the inappropriate behavior might have on others. To recognize the upcoming error, the subject has to anticipate the actor’s behavior on the basis of previously internalized experiences [19]. Although the questions posed in the RMET require somewhat less predictive ability, successful performance on the RMET also requires the subject to identify underlying intention and complex mental states from observing actors’ eye regions in a situation in which no other contextual cues are available, suggesting that advanced SC ability is required.

Conversely, good performances were observed when stories that required a minor level of prediction and error monitoring, such as the “no-faux pas” stories, were presented. Indeed, in these conditions, the social situation is univocal and is well described in the story text. This is also the case in the EA test, in which altered performance was only observed in the ASD population. EA requires proper processing of emotional impact based on coherent expectations about social interactions. Consistent with this, a key social impairment of individuals with ASD is related to the processing of emotions [105]; these individuals typically fail to accurately report others’ emotions [105] and to derive emotional meaning in social contexts [106].

Additionally, BD2 patients show a distinctive impairment in the faux pas affective component. While the cognitive component of ToM refers to the ability to infer others’ perspectives, feelings, and thoughts [107], affective ToM (i.e., inferring emotions) is known as the ability to feel what others are feeling by inferring emotions through cognitive perspective-taking [95,107,108]. Accordingly, existing studies have revealed that patients with BD exhibit abnormalities in both cognitive and affective processes associated with ToM [39]. Despite these findings, no significant differences were detected when the three groups of patients were directly compared, suggesting that CB, ASD, and BD2 patients had comparable SC performance.

Importantly, we found that the SC alterations observed were largely consistent with MRI findings. Indeed, in terms of structural alterations in the cerebellum, distinct and common patterns of regional decreases in cerebellar GM were found in CB, ASD and BD2 individuals compared to controls.

As expected, CB patients showed a more pronounced pattern of atrophy involving both the anterior (lobules I-V) and posterior (Crus I-II) cerebellar regions, while only the posterior (Crus I-II) and vermal regions (vermis Crus II) were affected in BD2 patients. Finally, selective involvement of the posterior cerebellum (Crus II) was detected in ASD individuals. Interestingly, a common pattern of cerebellar GM alterations in the right Crus II was evident in the three groups. Furthermore, direct comparison of the three groups revealed the presence of a diffuse cerebellar GM decrease involving the anterior and posterior cerebellar motor regions (I-V, VIIIa and VIIIb) only in CB patients, while no significant differences were found among the three groups in the amount of GM reduction in cerebellar Crus II.

The proven involvement of the “limbic cerebellum”, i.e., the cerebellar vermis, in emotional processing has been a crucial starting point for broadening our understanding of its role in affective and social functioning [53]. Advanced clinical, experimental, neuroimaging, and neurophysiological technologies have made it possible to conduct innovative lines of research on the emotional cerebellum, and consensus has emerged about its inclusion in the corticolimbic networks subserving emotion processing [53]. Starting with emotional perception and recognition, research then proceeded through the evaluation of more sophisticated aspects of social cognition, such as ToM [53]. Indeed, participation of the cerebellum in more advanced mentalizing functions has been confirmed by several neuroimaging studies [28,29,109] showing that social mentalizing recruits closed-loop circuitry between the posterior cerebellar Crus II and the temporoparietal junction (TPJ) and the medial PFC, two key areas of the mentalizing network [29,109].

Consistent with the evidence that more advanced ToM abilities are impaired in the CB, BD2, and ASD groups, a partial overlap between the two populations in terms of altered cerebellar regions has been found to specifically involve the right Crus II. Several fMRI connectivity studies [29,109] support the existence of a strong link between the posterior cerebellum, in particular Crus II, and key mentalizing areas in the cerebral cortex, including the TPJ and prefrontal regions.

fMRI studies have broadly proved that the cerebellum has a functional topographic arrangement, in the sense that function-specific cerebellar networks are coupled to function-specific networks in the cerebrum [24]. Consistently, the Crus II has been linked to an extensive spectrum of functional domains, other than mentalizing and ToM [110,111]. Most importantly, recent advances have strongly confirmed a central role for the posterior cerebellar Crus in identifying and automatizing action sequencing during social mentalizing and in predicting future action sequences based on social mentalizing inferences made about others [19,20,112,113]. Sequence processing has been proposed to be the basic cerebellar functional mechanism in both the motor and cognitive domains, leading to postulation of the “sequence detection theory” [114]. According to the sequencing hypothesis, the cerebellum detects and simulates repetitive patterns of temporally or spatially structured events regardless of whether they constitute sensory consequences of one’s actions in motor planning, expected sensory stimuli related to perceptual prediction, or inferences made through higher-order processes (e.g., cognitive elaboration or social cognition). Together, these findings support the view that the posterior cerebellum (i.e., Crus II) builds internal action models of our social interactions that are used to predict how other people’s actions will be executed and what our most likely responses to these actions will be [13]. It must be underlined that, according to the findings of the present work, a domain-specific mentalizing connectivity between the cerebrum and the cerebellum has been shown to specifically involve the Crus II of the right cerebellar hemisphere [28,29,109].

Overall, the present data indicate that in individuals with cerebellar damage, performance subtending social interactions becomes less accurate mainly when the stimuli require automatic processing or a high level of prediction, as in the RMET and Faux Pas tests, respectively. It can be hypothesized that the presence of specific cerebellar damage alters the modulatory function that is normally exerted by the cerebellum on its cortical projection areas and thereby affects the functional activity of key brain areas involved in various aspects of social cognition and, in particular, in the mentalizing process.

Specifically, the present preliminary results suggest that the common pattern of SC impairment observed in the three clinical groups in this study might be ascribed to impaired modulation by the posterior cerebellum, i.e., Crus II, of cerebral projection areas that are involved in more advanced social functions [19,50,109].

It must be mentioned that when the patterns of cerebellar structural reduction in the three groups were compared, no significant difference was found except in the case of the CB patients, who presented more pronounced cerebellar atrophy (with respect to both BD2 and ASD). This atrophy, however, was mainly localized to cerebellar motor areas. This is consistent with the existing evidence that motor problems are central in individuals with cerebellar neurodegenerative pathologies [115]. Interestingly, the significantly different pattern of changes in cerebellar GM observed in the CB patients as compared to the other clinical groups does not involve the cerebellar Crus II.

Generally, the role of the cerebellum may be explained as a prediction mechanism that is exerted within different cortico-cerebellar and cerebellar limbic networks involved in social cognition [112].

Imaging studies conducted in healthy subjects have demonstrated that cognitive and affective components of ToM recruit various neural systems. In particular, the dorsolateral PFC [116] is thought to play a role in inferring the cognitive mental states of others (i.e., cognitive ToM), whereas the ventromedial PFC is involved in making inferences about other people’s feelings and emotions (i.e., affective ToM) [108,117]. Accordingly, the distinctive impairment of ToM affective components in BD2 patients is consistent with the evidence that the ventromedial PFC is typically altered in this population and that it is mainly related to the rapid cycling that characterizes BD2 compared to BD1 [118,119]. In particular, prominent abnormalities in the ventromedial PFC-amygdala neural system are thought to play a key role in affective dysregulation of BD via its connections with other structures, including the cerebellum [120]. Interestingly, in the present study, GM reduction in the BD2 population was also found in the posterior cerebellar vermis (i.e., vermis Crus II), a region that is known to subserve affective processing through extensive reciprocal connections with other anterior limbic systems, including the prefrontal areas and the amygdala [121,122].

This is the first study to explore the similarities in SC profiles and in cerebellar GM in CB, ASD, and BD2 populations. As postulated in the “sequences hypothesis” [114], the present findings suggest that the cerebellum may play a crucial role in the social domain by detecting predictable sequences (i.e., by making and adapting internal models of social actions) and thereby providing the optimized feedforward control that is necessary to accomplish these functions in a fluid and automated manner [114,123]. Thus, cerebellar dysfunction involving key mentalizing regions, i.e., Crus II, may interfere with use of the internal model and thus prevent the prediction function and the making of correct inferences about the mental states of others, impacting the recognition of deviance from expected social behaviors.

Some limitations need to be mentioned. First, due to the heterogeneity of clinical samples and to the retrospective nature of the data, many key variables across groups could not be strictly controlled. In this regard and in agreement with the current state of the art on the psychopathological manifestation of BD and ASD, future studies are required to take into account more specific shared and distinctive features across these clinical samples, such as the presence of autistic traits in BD and suicidal risk in ASD individuals. Secondly, due to heterogeneity and small sample sizes of the groups, the present exploratory study does not provide evidence of a direct link between the observed changes in GM in the cerebellum and the reported performances on social tasks. Due to the relatively small sample sizes and since there was not a linear relationship between the performances at the SC tests and the GM parameters, we could not run regression analysis. Thus, the lack of regression analysis does not allow us to confirm the direct relationship between the gray matter reduction in the cerebellar right Crus II and the behavioral measures of social function in clinical groups. 

Furthermore, due to the difficulty of finding unmedicated BD patients in remission, it must be considered that the patients’ use of pharmacotherapy could have affected the changes in cerebellar GM observed in this population, as reported in a previous study [124]. However, later findings have disproven this evidence by reporting no effect of pharmacological treatment on cerebellar structures [125].

Despite the exploratory nature of the study, the present preliminary findings point to critical advances in current knowledge about the role of the cerebellum in the social domain, providing initial evidence regarding significant overlaps in terms of SC performances and cerebellar structural changes in different psychopathological conditions. However, these results may have not only theoretical significance but also a strong clinical impact. The importance of bringing “social cognition” into the clinical field is a current issue in the scientific community.

In this framework, elucidating the presence of common cerebellar structural abnormalities and SC impairment in different populations represents a further step in the effort to dissect the cerebellar topography related to the social domain, a goal that has important transdiagnostic impact in terms of novel alternative therapeutic indications in different clinical settings. To further address this issue, future hypothesis-testing studies specifically assessing the causal link between cerebellar alterations and social cognitive deficits across different clinical populations are needed and may help to also clarify whether cerebellar predictive mechanisms are central to the SC domain. Further insight can be gained by using a resting-state fMRI approach to assess whether a common or distinctive pattern of functional connectivity alterations underlies SC impairment in individuals with CB, ASD, and BD2. Indeed, considering that the nature and degree of impairments in social cognition are similar in individuals with different pathological conditions, the neural circuits that underlie these similar social impairments may be the same. In this framework, the identification of cerebello-cerebral circuits underlying specific social and emotional abilities may be fundamental for the development of transdiagnostic markers of social impairments.

## Figures and Tables

**Table 1:** Demographic characteristics and clinical scores of the studied groups.

Group	N	Sex F/M	Age Mean (SD)	Education Mean (SD)	Raven’s 47 Mean (SD)	IQ Mean (SD)	ICARS Mean (SD)	AQ Mean (SD)	HDRS Mean (SD)	YMRS Mean (SD)
**CB**	32	17/15	46.71 (11.03)	13.4 (3.45)	29,29 (2,9)	8662 (131)	2626 (126)	-	-	-
**ASD**	21	10/11	26.85 (8.66)	13.76 (1.72)	-	106.68 (20.7)	2.46 (3.8)	34.5 (9.9)	-	-
**BD2**	13	6/7	45.07 (11.22)	14.53 (3.79)	27.75 (7.75)	-	1.08 (2.66)	-	2.62 (3.23)	1.77 (2.74)
**HS-SC**	57	33/24	36.49 (13.15)	13.78 (2.73)	3120 (3.1)	108.29 (10.8)	-	16.3 (6.6)	-	-

Raven’s 47: cut-off =< 18.96; CB = cerebellar patients; ASD: autism spectrum disorder; BD = bipolar disorder; HS-SC = Healthy Subjects; N = Number; F = Female; M = Male; IQ = Intellectual Quotient (WAIS-R) [90,91]; ICARS = International Cooperative Ataxia Rating Scale [89]; AQ = Autism Quotient [92]; HDRS = Hamilton Depression Rating Scale [86]; YMRS = Young Mania Rating Scale [87].

**Table 2:** Means and standard deviation of scores obtained in each SC test by each group.

Group	RMET	EA	“Faux Pas” Stories	“No-Faux Pas” Stories	Affective	Cognitive
**CB**	21.77 (5.14)	40.10 (6.00)	38.77 (10.77)	19.53 (0.73)	6.30 (2.38)	32.46 (8.81)
**ASD**	21.14 (6.11)	37.57 (8.98)	39.33 (13.10)	19.57 (0.98)	5.71 (2.49)	33.62 (10.99)
**BD2**	22.00 (5.40)	43.10 (4.82)	30.60 (13.55)	19.60 (1.27)	4.50 (2.64)	26.10 (11.42)
**HS-SC**	26.37 (3.19)	43.86 (5.77)	47.12 (9.57)	19.19 (1.77)	6.56 (2.08)	40.56 (8.20)

The values are reported as mean and SD of scores obtained in the EA test (max = 58), in the RMET (max = 36), in the “Faux Pas” Stories (max = 60), in the “No-Faux Pas” Stories (max = 20), in the Cognitive Component (max = 50) and in the Affective Component (max = 10). Abbreviations: HS-SC, healthy subjects; CB, degenerative cerebellar damage; ASD, autism spectrum disorders; BD2, bipolar disorders type 2.

**Table 3:** Two-sample t-test showing regions of cerebellar GM reduction in CB, ASD and BD2 compared to controls.

Group	Size	Regions	Side	Coordinates (mm)			Peak Z-Scores	Peak-Level *p* Value (FWE)
				x	y	z		
**CB**	17,013	Lobule I–IV	L	−7	−37	−19	5.94	0.000
		Lobule I–IV	R	12	−37	−22	5.82	0.000
		Lobule VI	R	22	−58	−14	4.84	0.004
	2731	Crus-II	L	−12	−86	−29	4.88	0.003
		Crus-I	L	−17	−79	−21	4.72	0.006
**ASD**	2702	Crus-II	R	12	−76	−39	4.28	0.026
**BD2**	2972	Lobule IX	R	3	−47	−60	4.26	0.007
		Crus-II	R	4	−61	−54	4.52	0.012
	3148	Crus-II	R	6	−79	−34	4.23	0.008

**Table 4:** Between-groups ANOVA showing cerebellar GM reduction in CB compared to ASD and BD2.

Group	Size	Regions	Side	Coordinates (mm)			Peak Z-Scores	Peak-Level *p* Value (FWE)
				x	y	z		
**CB < ASD**	2131	Lobule VI	R	24	−59	−27	4.64	0.002
		Loule V	R	17	−50	−22	4.22	0.013
		Lobule I–IV	R	12	−38	−22	4.18	0.015
	1332	Lobule VI	L	−26	−52	−25	4.51	0.004
			L	−14	−57	−23	4.08	0.021
**CB < BD2**	26,163	VIIIb	R	20	−53	−45	6.42	0.000
		Lobule VI	L	−21	−52	−24	5.61	0.000
		Lobule V	R	18	−48	−23	5–60	0.000

## Data Availability

The data presented in this study are available on request from the corresponding author on reasonable request.

## Supplementary Materials

The following supporting information can be downloaded at: https://www.mdpi.com/article/10.3390/biomedicines11020309/s1, Table S1: Current pharmacotherapy of BD2 patients; Table S2: Results of pairwise comparisons between HS-SC, CB, ASD, and BD2, in the Social Cognition tests.
