# Supplementary material for: The Cerebellum Gets Social: Evidence from an Exploratory Study of Cerebellar, Neurodevelopmental, and Psychiatric Disorders
Source: Biomedicines. 2023 Jan 22;11(2):309. doi: 10.3390/biomedicines11020309 (PMC9953169; doi:10.3390/biomedicines11020309)
Supplement: Supplementary file 1 [file biomedicines-11-00309-s001.zip › biomedicines-2127946-supplementary.pdf]

Table S1. Current pharmacotherapy of BD2 patients.

Group	Antipsychotics	Lithium	Antiepileptics	Antidepressants	Anxiolytic	Polypharmacy
BD2	7	7	10	1	3	8

Number of BD patients taking the medication. Polypharmacy = Patients taking at least two different types of medication.

Table S2. Results of MANCOVA and pairwise comparisons between HS, BD2, CB and ASD in the Social Cognition tests.

MANCOVA	RMET	EA	FP stories	No-FP stories	Cognitive Comp.	Affective Comp.
Group effect	0.000*	0.001*	0.000*	0.581	0.000*	0.054*
Pairwise comparisons						
CB vs. HS-SC	0.001*	0.159	0.012*	1.00	0.003*	1.00
ASD vs. HS-SC	0.000*	0.002*	0.054*	1.00	0.033*	1.00
BD2 vs. HS-SC	0.054*	1.00	0.000*	1.00	0.000*	0.051*
CB vs. ASD	1.00	1.00	1.00	1.00	1.00	1.00
CB vs. BD2	1.00	1.00	0.272	1.00	0.374	0.239
ASD vs. BD2	1.00	0.163	0.301	1.00	0.307	0.851

*Results significant at $p < 0.05$; pairwise comparisons were adjusted using the Bonferroni correction for multiple comparisons.
